# Thoracic pseudotumours: a pictorial essay

**DOI:** 10.1186/1470-7330-15-S1-P26

**Published:** 2015-10-02

**Authors:** S De Luca, C Carrera, A Zanfardini, L Tolkachier, D Pascuzzi, E Eyheremendy

**Affiliations:** 1 Hospital Aleman, Buenos Aires, Argentina

## Learning objectives

Imaging appearance of primary lung tumour conditions that on initial radiological studies might be confused with malignant lesions.

## Content organisation

Diagnostic tools for evaluation of thoracic tumours and pseudotumours. We included in the differential diagnosis:

Paraffinoma (1), a hydatid cyst (1), inflammatory pseudotumour (3), nodular tuberculosis (2) and sarcoidosis granulomas (1), round pneumonia (1), nodular criptococosis (1), post-surgical or tuberculosis scarring processes (2), organising cryptogenetic pneumonia (2) and round atelectasis (3).

## Conclusion

There are several diseases that can mimic tumours of the chest. It is necessary to take them into account and know how to use diagnostic keys for an accurate diagnosis.

